# Diffusion tensor imaging in the musculoskeletal and peripheral nerve systems: from experimental to clinical applications

**DOI:** 10.1186/s41747-017-0018-1

**Published:** 2017-09-30

**Authors:** Vito Chianca, Domenico Albano, Carmelo Messina, Claudia Maria Cinnante, Fabio Maria Triulzi, Francesco Sardanelli, Luca Maria Sconfienza

**Affiliations:** 1 Department of Advanced Biomedical Sciences, Università Federico II, Via Pansini 5, 80131 11 Napoli, Italy; 2 Department of Radiology, DIBIMED, Università di Palermo, Via del Vespro 127, 90127 Palermo, Italy; 3 Unit of Neuroradiology, Fondazione IRCCS Ca’ Granda Ospedale Maggiore Policlinico, Via Francesco Sforza 35, 20122 Milano, Italy; 4 Unit of Radiology, IRCCS Policlinico San Donato, Via Morandi 30, 20097 San Donato Milanese, Italy; 5 Department of Pathophysiology and Transplantation, Università degli Studi di Milano, Via Festa del Perdono 7, 20122 Milano, Italy; 6 Department of Biomedical Sciences for Health, Università degli Studi di Milano, Via Mangiagalli 31, 20133, 20122 Milano, Italy; 7 Unit of Diagnostic and Interventional Radiology, IRCCS Istituto Ortopedico Galeazzi, Via Riccardo Galeazzi 4, 20161 Milano, Italy

**Keywords:** Magnetic resonance imaging, Diffusion tensor imaging, Tractography, Muscle, Tendon

## Abstract

Magnetic resonance imaging (MRI) is a well-established imaging modality which is used in all districts of the musculoskeletal and peripheral nerve systems. More recently, initial studies have applied multiparametric MRI to evaluate quantitatively different aspects of musculoskeletal and peripheral nerve diseases, thus providing not only images but also numbers and clinical data. Besides ^1^H and ^31^P magnetic resonance spectroscopy, diffusion-weighted imaging (DWI) and blood oxygenation level-dependent imaging, diffusion tensor imaging (DTI) is a relatively new MRI-based technique relying on principles of DWI, which has traditionally been used mainly for evaluating the central nervous system to track fibre course. In the musculoskeletal and peripheral nerve systems, DTI has been mostly used in experimental settings, with still few indications in clinical practice. In this review, we describe the potential use of DTI to evaluate different musculoskeletal and peripheral nerve conditions, emphasising the translational aspects of this technique from the experimental to the clinical setting.

## Key points

- Diffusion tensor imaging (DTI) is feasible in the musculoskeletal and peripheral nervous systems
- Most studies on musculoskeletal DTI were performed in experimental settings
- Clinical data on DTI in the musculoskeletal system are still sparse
- Standardisation of protocols in musculoskeletal DTI is a major issue
- Quantitative evaluation of musculotendinous damage is the most promising clinical application

## Introduction

Magnetic resonance imaging (MRI) is a well consolidated modality in all districts of the musculoskeletal system. Until a few years ago, most musculoskeletal and peripheral nerve disorders were qualitatively assessed with MRI using standard morphological sequences [1]. More recently, initial studies have applied multiparametric MRI to quantitatively evaluate different aspects of musculoskeletal and peripheral nerve diseases, thus providing not only images but also numbers and clinical data [2]. ^1^H and ^31^P magnetic resonance spectroscopy have been used for soft-tissue lesion characterisation [3], to measure muscular fat content [4] and to evaluate the effect of physical exercise in muscles [5]. Diffusion-weighted imaging (DWI) has been applied mainly to differentiate benign from malignant lesions [6]. Blood oxygenation level-dependent imaging has been used for evaluating muscle structural and functional changes [7].

Diffusion tensor imaging (DTI) is a relatively new MRI-based technique relying on principles of DWI, which has traditionally been used mainly for evaluating the central nervous system to track fibre course [8]. In the musculoskeletal and peripheral nerve systems, DTI has been mostly used in experimental settings, with still few indications for clinical practice.

In this review, we describe the potential use of DTI to evaluate different musculoskeletal and peripheral nerve conditions, emphasising the translational aspects of this technique from the experimental to the clinical setting.

## Technical considerations

### The concept of anisotropy and water diffusion

The principle of water molecule diffusion evaluated using MRI in the human body was firstly reported in 1985 [9] to assess microstructural changes in fibre architecture involved in pathologic conditions of the central nervous system [10]. Molecular diffusion refers to the random translational motion of molecules, also called Brownian motion, as a result from the thermal energy they carry [11]. Diffusion can be studied by using spin-echo single-shot echo-planar imaging sequences [12] with adequate fat suppression. DWI sequences have technical parameters not different from those of conventional sequences, such as time of echo (TE) and time of repetition (TR), but also an additional parameter, i.e. the b-value [11]. This parameter represents the degree of diffusion weighting of the sequence, determined by the application of specific magnetic field gradients, and is measured in s/mm^2^. The amount of water molecule diffusion is quantified by the apparent diffusion coefficient (ADC)—obtained by interpolating the results given with different applied b-values—which provides indirect information also about the arrangement of the surrounding structures.

Molecular mobility in human tissues is usually non-isotropic, which means that diffusion does not occur equally in all directions; protein fibres, cell membranes and myelin sheath tend to hinder water diffusion [13].

The effect of diffusion anisotropy can be easily detected by evaluating variations in the diffusion measurements when the direction of gradient pulses is changed [14]. Fractional anisotropy (FA) is a parameter used to quantify the directional orientation of water molecules within the tissue. FA values are in the range of 0–1. When a tissue is intact, water is forced to move in a specific direction and the FA value is close to 1. When tissues have micro- or macro-structural damages, the water molecules are directed in multiple directions and the FA value decreases toward 0 [15].

DTI is derived from the concept of water molecule diffusion. In DTI, it is possible to calculate FA and evaluate the preferred spatial movement of molecules. Hence, since structural arrangement makes the water diffusion prevalent along the major axis of fibrillary tissues, DTI can be used for fibre tractography. To calculate the diffusion tensor, we need to acquire DWI with high b-values along at least six non-collinear directions in addition to a low b-value DWI or a T2-weighted sequence [16]. The higher the number of directions along which the diffusion gradients are applied, the higher the accuracy of anisotropy calculation. Clearly, increasing the number of directions implies an increase in scan times.

To optimise the study protocol, the spatial complexity of the structure under investigation should be considered. Acquisition time is largely variable, as standardised protocols are still lacking. As an example, six directions may be enough for the median nerve, about 15–25 vector directions are needed for the brachial plexus, while 10–12 different vector directions are needed for muscular structures [13]. Regarding the b-values, there is no unanimous agreement on what to use in musculoskeletal imaging [17]. Higher b-values increase the power of the gradients and diffusion weighting of the sequence, but reduce the signal-to-noise ratio (SNR). Oudeman et al. suggest optimising the SNR using voxel volumes in the range of 20–30 mm^3^, short TE, b-values in the range of 400–500 s/mm^2^ and at least ten gradient directions [18]. Clearly, the higher the B_0_ magnetic field strength, the higher the SNR [19]. However, Alexander et al. reported that FA and ADC do not significantly change in correlation with field strength [20].

### Tractography

**Fig. 1:**
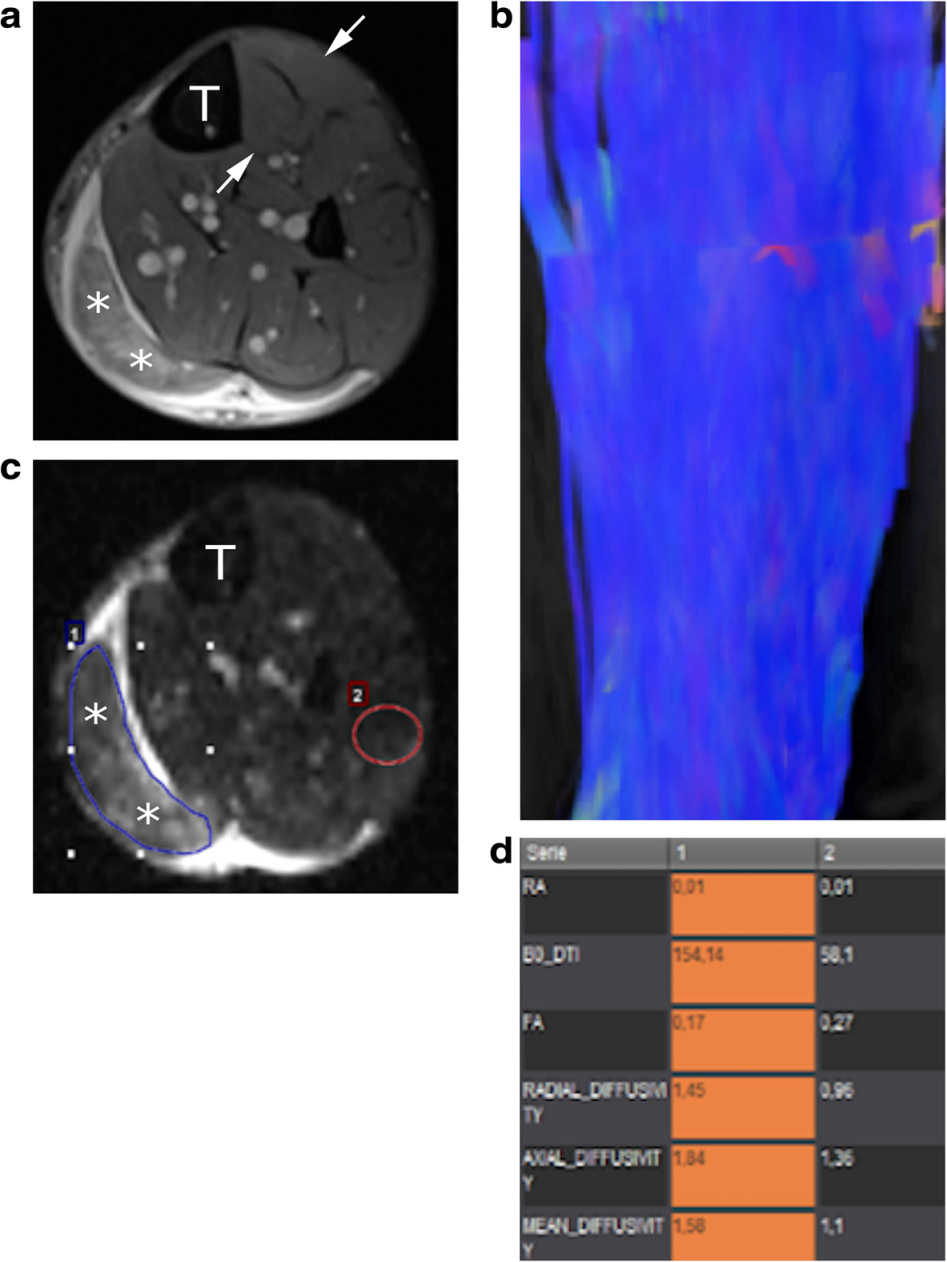
**a** Axial fat-saturated proton-density image of the middle third of the leg of a 29-year-old male patient who sustained a grade 2c tear of the medial gastrocnemius muscle (*asterisks*). *T* tibia, *arrows* normal tibialis anterior muscle. **b** Tractography of the tibialis anterior muscle, showing normally oriented and arranged fibres. **c** Regions of interest are placed on the torn muscle (1) and healthy muscle tissue (2). **d** Quantitative analysis shows different values in the torn muscle compared to healthy tissue, in particular a lower FA (0.17) compared to that of healthy fibres (0.27)

Tractography represents an application of DTI [21] that allows the analysis of muscle architecture aspects such as pennation angle, curvature of fibres, fibre length and possible muscle fibrosis (Fig. 1) [22]. Generally, muscle fibres are aligned parallel and do not diverge or curve as myelin sheath tracts; for this reason, it is possible to use less gradient directions than in the study of nervous structures. Accurate FA maps are necessary to manually or automatically draw a region of interest within the muscle. The features of the analysed tract may depend on some intrinsic muscles properties such as fatty infiltration, muscle atrophy, fibres tracking algorithm setting and the possible presence of partial volume artefacts [23].

## Specific applications in the musculoskeletal system

### Experimental applications

First applications of DTI in the evaluation of muscle architecture were validated on small animals [24]. Damon et al. evaluated the pennation angle of muscles on animal models, finding a high correlation (r = 0.89) between angles measured with DTI and those measured by direct anatomical inspection [25]. Zhou et al. monitored chick embryonic skeletal muscle development in ovo (during incubation days 5–18 under a 3 T scanner) and investigated the correlation between FA and fibre length, finding that the result of DTI-tracked fibres during incubation corresponds to the development of chick embryonic skeletal muscle [26]. McMillan et al. [27] found increased axial and radial diffusivity on ADC maps and decreased FA of muscle in dystrophic wild-type mice vs. normal controls.

### Normal muscle tissue

Feasibility of DTI in the evaluation of normal muscle tissue has been widely demonstrated, with high reproducibility [16]. In different studies, capability of DTI in evaluating muscle cross-sectional area, fibre length and pennation angle has been demonstrated. Some studies demonstrated feasibility of DTI to assess changes of fibre orientation according to positional variations of different body segments [28]. DTI was also used to measure fibre orientation, which has been reported to be a parameter that potentially predicts the pattern of tearing after muscle strain and evaluates the presence of a muscular lesion. An example of DTI of normal muscle tissue is reported in Fig. 1a and b.

### Muscle contraction and muscle injury

After physical exercise, the contractile complex of muscle, composed of actin and myosin, increases in size and density. This hypertrophic response of muscular tissue determines an increase in the thickness of endomysium muscle wrap and sarcoplasmic reticulum [29]. Okamoto et al. studied two volunteers using DTI immediately, 24 h, 48 h, and one week after unilateral exercises of repeated flexion and extension of the ankle with loading. They reported a decrease of FA values in the posterior muscle bellies of the gastrocnemius and soleus muscles in comparison with FA values of the contralateral calf muscles immediately after exercise [30]. Froeling et al. studied the upper legs of five male amateur long-distance runners one week before and two days and three weeks after a marathon. They found that FA and mean diffusivity (MD), an index that reflects the average magnitude of molecular displacement by diffusion, were significantly increased in the biceps femoris muscle; also, MD was significantly increased in the semitendinosus and gracilis muscles two days after the marathon, while there were no changes on fat-suppressed T2-weighted sequences [31]. Zaraiskaya et al. [32] studied four patients with gastrocnemius and soleus muscles injuries comparing them to eight healthy controls. Authors found significant differences in FA and ADC values in injured skeletal muscle which presented very low values (0.08 ± 0.02 vs. 0.23 ± 0.02 in healthy controls). Since diffusivity increases only in the muscles that are more injured after running, these data imply that DTI parameters might become a powerful diagnostic tool for prognosis and response assessment to treatment of sports-related muscle injuries (Fig. 1a, c, d). Further future application of DTI might be the monitor of restitution ad integrum of torn muscles in athletes.

### Muscular dystrophy

Under the name of muscular dystrophy, a group of genetic, progressive and degenerative muscular diseases is included, whose primary symptom is muscle weakness [33]. These pathologic conditions may occur at any age with variable clinical features [34]. The early-onset muscular dystrophies may be associated with profound loss of muscle function, affecting ambulation and posture, and may lead to respiratory and cardiac impairment. Conversely, late-onset muscular dystrophies or myopathies may be less symptomatic and associated with slight weakness and inability to increase muscle mass [34].

**Fig. 2:**
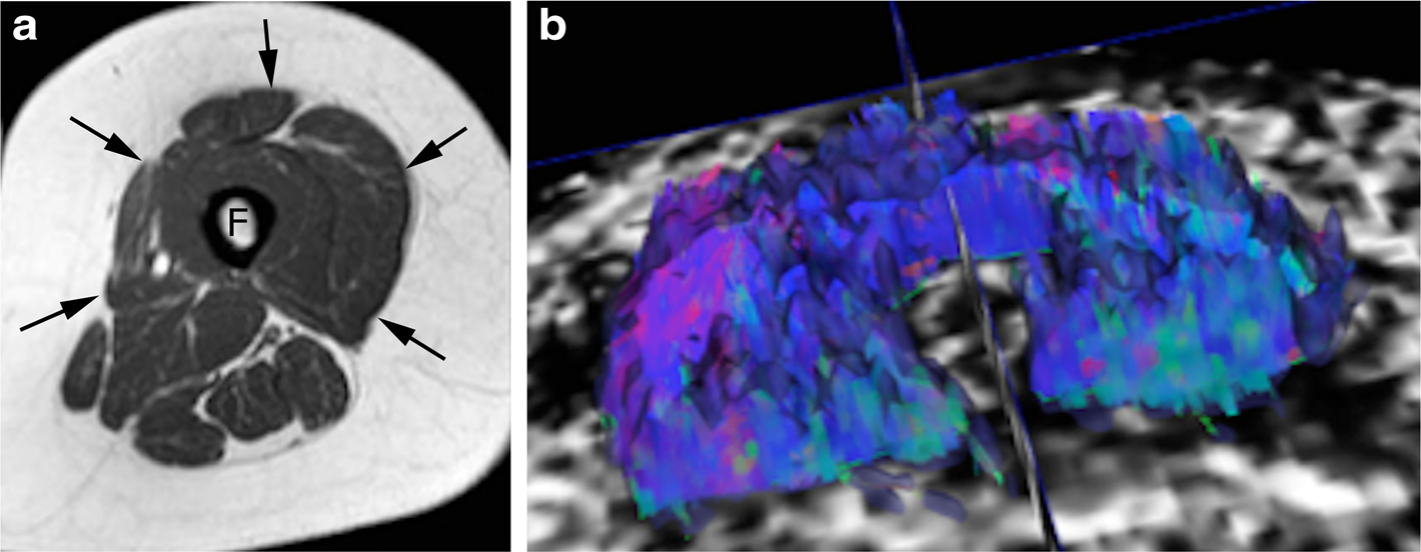
Magnetic resonance of the thigh performed on a 42-year-old male aptient with mild limb-girdle muscular dystrophy. **a** Morphological appearance on axial T1-weighted image (*arrows*). *F* femur. **b** The corresponding tractography image shows fibres which are decreased in number, length and organisation due to partial fatty replacement. This appearance can be particularly appreciated if compared to Fig. 1b, which shows a normal participant

Duchenne muscular dystrophy (DMD) is the most common form of inherited muscular dystrophy in children. It is caused by mutations in the X-linked dystrophin gene and is characterised by systemic muscle weakness due to the progressive destruction of skeletal muscle [35]. In this setting, quantitative evaluation of muscle status may be very important, both to reliably aiding the diagnosis and to monitor the effect of treatments [33]. Until a few years ago, disease progression was only evaluated quantitatively on MRI. Mercuri et al. developed a four-point grading system based on fatty tissue infiltration to categorise disease severity [36]. Ponrartana et al. [35] focused their attention on possible quantitative MRI parameters to monitor the disease progression in the lower extremities of boys with DMD and found a strong correlation between DTI values, clinical test and qualitative evaluation using the Mercuri scale [36]. Unexpectedly, they found patients with more severe DMD positively correlated with FA values and negatively with ADC values, while muscle strength negatively correlated with FA and positively with ADC values. One possible explanation of these results is the artificial decrease of ADC and artificial increase of FA values in participants with more than 45% fat muscle infiltration [37]. Conversely, tractography images showed the expected decrease in fibre length, number and architecture, suggesting that fibre tracking may provide further quantitative information (Fig. 2). Li et al. [38] showed that damage to thigh muscles in DMD patients can be evaluated by ADC and FA values and can be applied to assess quantitatively disease severity.

### Ligaments

**Fig. 3:**
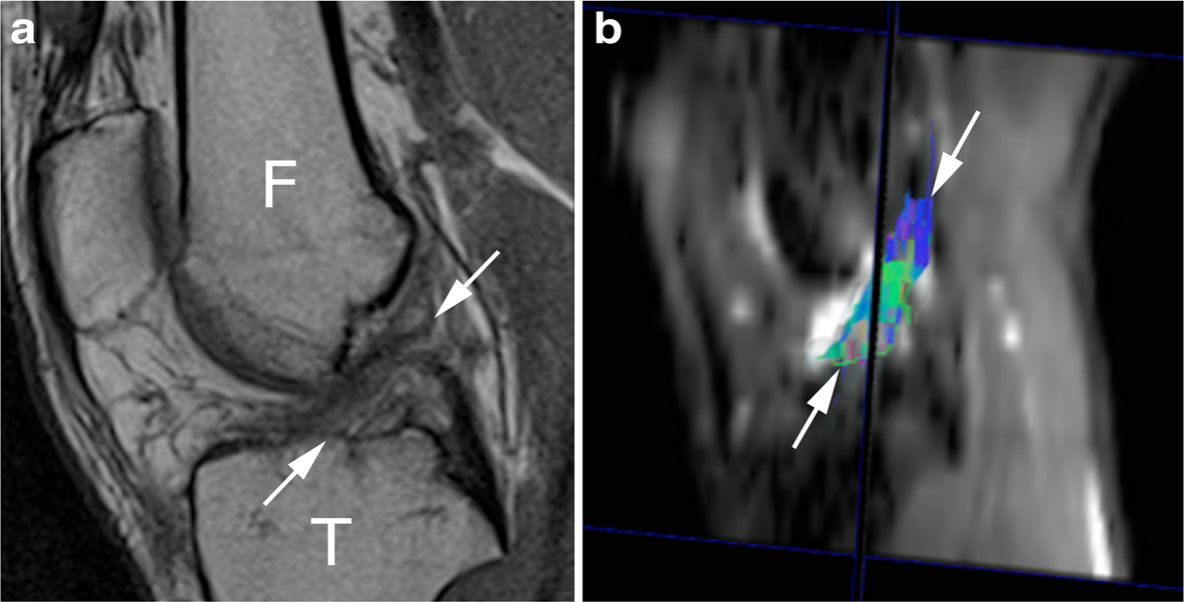
Magnetic resonance of the knee performed on a 24-year-old male patient two months after soccer injury. **a** Sagittal T1-weighted image shows that the anterior cruciate ligament is remarkably inhomogeneous due a partial tear (*arrows*). *F* femur, *T* tibia. **b** The corresponding tractography image shows that fibres are partially interrupted and architecture is disrupted (*arrows*)

Arthroscopic grafting for ligament tears is common in active population [39] and MRI is routinely used to assess graft integrity. Despite the high diagnostic performance of conventional MRI [40], it cannot allow advanced quantitative evaluation. Graft ligaments progress through four stages: avascular necrosis; revascularisation; cellular proliferation; and remodelling [41], with different signal intensities at conventional MRI. The potential use of DTI was firstly reported by Yang et al. [42], who performed DTI sequences in 40 healthy volunteers and in 15 patients with anterior cruciate ligament (ACL) reconstruction. He found that ACL grafts in different stages present different FA and ADC values, with significantly higher FA value at ten years from surgery. More recently, Van Dyck et al. [43] found lower FA and higher MD values of ACL graft in comparison to the study by Yang et al. [42]. Differences could be related to a different reconstruction software and a shorter time between surgery and MRI [43]. Indeed, after four months, thick synovial tissue starts to envelop the graft providing its vascular supply [44]. Future research may be aimed to test feasibility and reliability of DTI to quantitatively assess the fibrillar structure, especially in case of a partially torn ligament (Fig. 3).

### Peripheral neuropathies

**Fig. 4:**
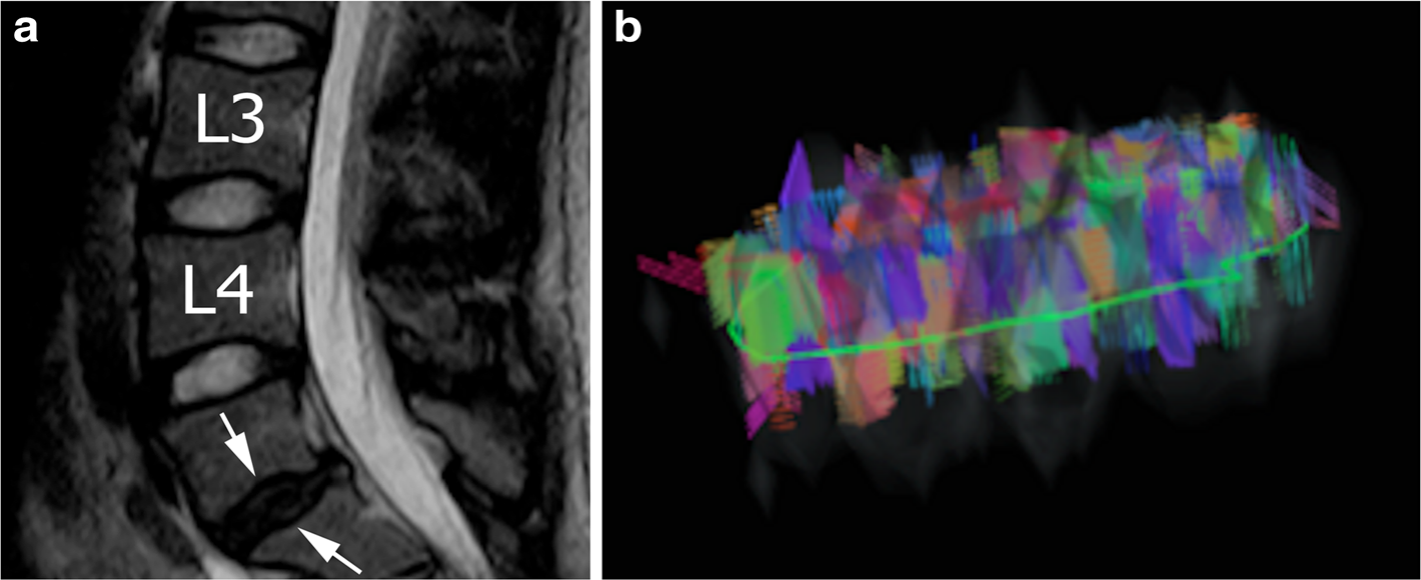
Magnetic resonance of the lumbar spine performed on a of 31-year-old man. **a** Sagittal T2-weighted image shows protrusion of a thinned and dehydrated intervertebral disc (Pfirrmann IV, *arrows*) at L5-S1. **b** Tractograpy shows irregular, disordered and thin fibres

Cervical spondylotic myelopathy (CSM) is the most common spinal cord disorder in patients aged > 55 years and it is seen in as many as 95% of men and 70% of women aged 60–65 years [45]. It is due to a chronic progressive compression of the cervical spinal cord (CSC) caused by degenerative disc disease, spondylosis or other degenerative pathology [46]. Although diagnosis of CSM is based primarily on clinical manifestations, MRI is routinely used to evaluate CSC, the canal size or the Torg-Pavlov ratio and potential signal changes of the compressed spinal tract [47]. However, all these findings are not always seen in symptomatic patients and there are often discrepancies between clinical and imaging features [48]. DTI and tractography can detect micro-architectural changes of CSC [29]. Studies reported an increase in MD values and a decrease in FA values around compressed CSC [49]. The diminished FA value seems to be a more sensitive parameter of cord injury than T2-weighted signal hyperintensity [50] and appears to be strictly correlated to symptom severity [51]. Tractography can detect abnormalities of the annulus fibrosus. The normal annulus has a consecutive and regular ring configuration composed by multilayer fibres, while the degenerative annulus shows irregular and disordered aspect (Fig. 4), becoming thinner and disrupted [52].

### Brachial plexus

Evaluation of brachial plexus abnormalities represents a diagnostic challenge due to anatomical complexity and to overlapping presentation of both benign and malignant conditions. There is a wide range of disease which may involve the brachial plexus, including tumours, radiation fibrosis, trauma and inflammatory processes. MRI is the imaging modality of choice for the evaluation of the brachial plexus [53]. To date, DTI of the brachial plexus at 3.0 T has been reported as feasible and reproducible in a series of healthy volunteers [54].

### Cubital tunnel syndrome

Cubital tunnel syndrome is the second most common peripheral compression neuropathy after carpal tunnel syndrome (CTS) [55]. It is related to a combination of compression and friction of the ulnar nerve in the cubital tunnel [56]. Breitenseher et al. [57] showed significant reduction of ulnar nerve FA values at the cubital groove and when passing the deep flexor fascia. At tractography, they showed complete or partial discontinuity of the ulnar nerve in 65% of patients. However, they conclude that T2 neurography is more sensitive than DTI in the detection of cubital tunnel syndrome [57].

### Carpal tunnel syndrome

CTS is the most common compressive peripheral neuropathy, affecting 3–4% of the general population [58]. It is caused by an entrapment of the median nerve at the level of the carpal tunnel of the wrist. The diagnosis of CTS is usually established by clinical features, clinical tests, electrophysiological tests, and high-resolution ultrasonography [59]. However, electrophysiology is associated with pain and conspicuous rate of false-negative and false-positive results [60].

Conventional MRI of CTS gives variable qualitative results [61]. DTI may potentially improve the diagnosis of peripheral nerve disorders, optimise lesion localisation, and determine the extent of neural dysfunction [62]. FA tends to increase distally in healthy participants and to decrease in patients with CTS for the presence of intrafascicular oedema. Kabakci et al. reported that FA values of two patients (FA = 0.41 and 0.44) were significantly lower than that of the control group (FA = 0.709 ± 0.046). Diffusion parameters are not influenced by the patients’ gender, while changes in diffusion and FA similar to those observed in CTS may appear with age, also without clinical symptoms [63]. Indeed, FA measured at the carpal tunnel inlet seems to have the highest accuracy for the diagnosis of CTS (sensitivity of 62%, specificity of 82%, positive predictive value of 80%, negative predictive value of 75%) [64] compared to other imaging modalities, and it showed the highest correlation with sensory and motor amplitude (r = 0.54, *p* < 0.001), even though it always needs to be correlated with clinical symptoms.

### Sciatic nerve and piriformis syndrome

Sciatic neuropathy is a common cause of lower extremity pain, which can be related to several causes affecting any level of the nerve and resulting in different symptoms [65]. The diagnosis is made through the patient history, clinical findings, and electrophysiological tests [66]. Routine MRI has been widely used to study the normal anatomy and pathology of the sciatic nerve and the associated muscle oedema or denervation, especially on T2-weighted sequences [67]. DTI may implement conventional MRI examination, providing additional information regarding the myelinic structures [68]. DTI does not show significant differences in either FA or ADC values at any level between sciatic nerve in the dominant or non-dominant lower limb. Wata et al. evaluated ten patients with sciatica symptoms, reporting lower FA and higher ADC values in the affected sciatic nerve compared with healthy controls [69].

Piriformis syndrome (PS) is a not easily recognisable disorder, characterised by buttock and leg pain due to compression of the sciatic nerve through or around the piriformis muscle in patients with usually normal neurological examination [70]. Manoeuvres of flexion, adduction and internal rotation of the hip, and direct palpation of the piriformis, cause severe pain. CT and MRI may help to diagnose PS and to differentiate it from other possible causes of lower lumbar pain; however, imaging diagnosis remains challenging [68].

### Nerve tumours

Previous studies reported higher frequency of destruction or massive fibre disorganisation in malignant peripheral nerve tumours, whereas benign tumours are often associated with dislocation or partial interruption of fibres. Chhabra et al. reported that tractography may provide three-dimensional visualisation of fibre dislocation or destruction. On the other hand, FA and MD show low values that indicate malignancy extension in neural structures [71].

## Conclusion

DTI and tractography may provide useful additional information allowing a quantitative analysis of healthy and pathological nerves, myelin sheaths, and muscles. To date, these tools are mainly applied in experimental settings and are uncommonly translated to clinical practice.

Since application of DTI and tractography in the musculoskeletal and peripheral nerve systems is different from application in the central nervous system, some issues still need to be addressed, such as: standardisation of acquisition protocols with optimised parameters and duration times; optimisation of post-processing software for DTI to be used in the musculoskeletal system; investigation of the possibility of using DTI around metallic implants, used in clinical practice more and more frequently; and analysis of reproducibility of DTI quantitative measurements (inter-study reproducibility; comparison among MR units from different vendors and between 1.5 and 3 T magnets; comparison among different DTI post-processing software).

At any rate, DTI and tractography seem to be promising tools and are able to provide useful quantitative information about muscular tissue and peripheral nerves as an adjunct to morphological MRI sequences. The definition of the real clinical added value of this approach requires well designed studies on large population samples, possibly with clinical end-points potentially showing a benefit to the patients which is not limited to diagnostic performance.

## Abbreviations

ACL: Anterior cruciate ligament
ADC: Apparent diffusion coefficient
CSM: Cervical spondylotic myelopathy
CTS: Carpal tunnel syndrome
DMD: Duchenne muscular dystrophy
DTI: Diffusion tensor imaging
DWI: Diffusion-weighted imaging
FA: Fractional anisotropy
MD: Mean diffusivity
MRI: Magnetic resonance imaging
PS: Piriformis syndrome
SNR: Signal-to-noise ratio
TE: Time of echo
TR: Time of repetition
